# Annotations

**Published:** 1906-01-13

**Authors:** 


### ANNOTATIONS.

### Kitchen Chemistry.

MOST people have a little of what is called science in their education. Sometimes this science is interesting, sometimes the reverse, but rarely is it of much practical value. But the study of chemistry is now being applied to the practical work of teaching girls how, with the simplest apparatus, they can test in their own homes whether or not many articles of food are adulterated. Thus, good butter can be distinguished from margarine by simply boiling a sample. Margarine boils noisily, sputtering like a mixture of grease and water, and with little or no foam. Genuine butter boils much more quietly and produces abundant foam. To test the presence of coal-tar dyes in such things as jam, fruit syrups, or any other very red article of food, all that is necessary is to boil a piece of white woollen cloth, first wet thoroughly with boiling water, in the suspected article for five or ten minutes, and then wash out the cloth in boiling water. The natural colouring of the fruit will leave the cloth only a dull pink in hue, while artificial dyes make it a brilliant red. Honey is often adulterated with glucose, and its presence can be discovered by the putting some of the honey in strong spirit of wine. If glucose is present it will cause turbidity in the spirit, and will settle at the bottom in a thick gummy mass, while genuine honey forms into a flocculent precipitate, and when it has settled leaves no turbidity. Adulterants are not always added because they are cheaper than the genuine article, but the public have a right to know when they are used; and it would be a distinct gain if, in case of suspicion, a simple test could always be applied.

### Juvenile Tobacco-Smoking.

THERE has been much loose talking upon this subject since Calverley wrote:—

How they who use fusees
All grow by slow degrees
Brainless as chimpanzees,
Meagre as lizards;
Go mad and beat their wives;
Plunge (after shocking lives)
Razors and carving-knives
Into their gizzards.

And yet there is wanting some authoritative and scientific pronouncement as to the actual harm done to his physique by the juvenile smoker. It is common to hear that smoking stunts the growth; it is constantly repeated as a fact, and that by medical men of eminence. But is there any truth in the charge? Certainly we do not recall that those of our school-fellows who lost no opportunity of smoking in their youth have grown up to be unsatisfactory citizens. There is room for some really careful observation on the effects of tobacco-smoking upon the economy, for there is little doubt that idiosyncrasy is particularly in evidence in connection with this drug. The smoker's heart, for instance, is a well-recognised physiological effect of excessive smoking—excessive, that is to say, for the individual. Syncopal attacks are also quite common; insomnia in a good number of people is a common sequel to any function which entails an unusual consumption of tobacco late at night. On the other hand, many men can smoke from sunrise to sunset for years, and inhale every mouthful of smoke, with never a symptom. This immunity, of course, gives them occasion to blaspheme those who suggest that the practice of smoking may be physically deleterious; and no progress is made towards the truth. We are far from upholding the practice of smoking in youth; if it is not harmful it is an expensive indulgence, often an excuse for idleness, and generally unsuited for the disciplinary years of boyhood. But this is no defence for labelling the practice "Poison" without some more definite evidence than has yet been brought forward.

### Sophisticated Silk and Poisoned Pencils.

MORE indignation than alarm has probably been excited among the fair sex by the statement that the skin is liable to become affected as a result of the manner in which silk is sophisticated. It is affirmed that the substances set free by the action of the perspiration upon certain metallic compounds now used in the manufacture of silk may have this effect. Of course, to avoid the possibility of ill results, ladies need only relinquish the practice of wearing silk, but we shall be surprised if they do not prefer to run the risk of skin disease to the alternative. That there is a measure of risk cannot be denied, assuming that the metallic compounds are used to the alleged extent of 36 per cent. We imagine, however, that there are manufacturers who are quite prepared to guarantee that their materials are absolutely free from adulteration of any deleterious character. A more disquieting statement comes from America. In a public school at Detroit there has been an epidemic which has been traced to the use of lead pencils. It has been the custom for the school managers to provide pencils for the pupils, gather them up in the evening, and distribute them indiscriminately again in the morning. One of the children contracted diphtheria, and in a few days there were 50 pupils down with the malady. Inquiries appeared to prove that the cases were mainly attributable to the habit on the part of the pupils of sucking their pencils, or holding them between their lips. The mischief being done, the authorities ordered the destruction of the pencils, and that each child should have, for the future, his own individual pencil. The system just abolished at Detroit does not, we believe, prevail in schools on this side of the Atlantic, but we think it desirable to emphasise the fact that it is perilous to allow children to exchange pencils. It may not be easy to prevent some from putting pencils into their mouths, but at least it is possible to hinder them from becoming agents for the communication of disease to others; and every child in every school should therefore be warned, on pain of an unpleasant penalty, not to lend or to borrow a pencil or a penholder lest it become a purveyor of poison.

